# Solid shrimp waste derived nanoporous carbon as an alternative bio-sorbent for oxytetracycline removal from aquaculture wastewater

**DOI:** 10.1016/j.heliyon.2024.e32427

**Published:** 2024-06-04

**Authors:** Napat Kaewtrakulchai, Nippit Samattakarn, Sirayu Chanpee, Pornsawan Assawasaengrat, Kanit Manatura, Sutthipoj Wongrerkdee, Apiluck Eiad-Ua

**Affiliations:** a Kasetsart Agricultural and Agro-Industrial Product Improvement Institute, Kasetsart University, Bangkok, 10900, Thailand; b Department of Chemical Engineering, School of Engineering, King Mongkut's Institute of Technology Ladkrabang, Bangkok, 10520, Thailand; c Department of Mechanical Engineering, Faculty of Engineering at Kamphaeng Saen, Kasetsart University, Kamphaeng Saen Campus, Nakhonpatom, 73140, Thailand; d Department of Physical and Material Sciences, Faculty of Liberal Arts and Science, Kasetsart University Kamphaeng Saen Campus, Nakhon Pathom, 73140 Thailand; e College of Materials Innovation and Technology, King Mongkut's Institute of Technology, Bangkok, 10520, Thailand

**Keywords:** Shrimp waste, Nanoporous carbon, Hydrothermal carbonization, Activation, Oxytetracycline, Adsorption

## Abstract

Recently, it has been critical to effectively remove oxytetracycline (OTC) from aquaculture wastewater before releasing into the environment. The adsorption process is recognized as an efficient pathway for removing OTC since it is a simple, stable, and cost-effective method. This study aims to develop nanoporous carbon entirely from shrimp waste (SW) via hydrothermal carbonization assisted with KOH activation. Existing KOH significantly increases the porosity of SW nanoporous carbon. The optimal SW porous carbon was obtained using 5 wt%KOH for activation, which had the largest surface area of 679.51 m^2^/g with the total pore volume of 0.458 cm^3^/g. Moreover, the SW porous carbon with the highest porosity was selected for the OTC adsorption. The Langmuir isotherm model and the pseudo-second-order kinetic model match the experimental data, implying that the adsorption mechanism is mono-layered adsorption due to micropores by chemisorption interaction. The adsorption capacity significantly improved by increasing the dosage of SW nanoporous carbon. The SW nanoporous carbon adsorption for OTC is primarily regulated by pore filling affected by hydrogen bonding, and π-π* interaction also plays a significant role. The SW nanoporous carbon showed an efficient OTC adsorption after 5 regeneration cycles. This work demonstrates biomass waste recycling and emphasizes the potential of aquatic food processing waste-derived nanoporous carbon for antibiotic adsorption.

## Nomenclature

**symbols**
SW: Shrimp waste
SWC: Shrimp waste derived nanoporous carbon
OTC: Oxytetracycline
HTC: Hydrothermal carbonization
S_BET_: BET specific surface area
V_T_: Total pore volume
V_mic_: Micropore Volume
V_mes_: Mesopore volume
D_p_: Average pore diameter
q_e_: Adsorption capacity at equilibrium
q_t_: Adsorption capacity at different time
C_i_: Initial concentration
C_e_: Equilibrium concentration
C_t_: Concentration at different time
V: Solution volume
M: Mass of adsorbent

## 1 Introduction

With the world's population growing rapidly, aquaculture has been promoted as a food security strategy to meet increased food consumption in numerous countries. Therefore, about 100,000 metric tons of antibiotics and veterinary medicines treat aquaculture diseases worldwide [1]. Oxytetracycline, chlortetracycline, and tetracycline are the principal aquaculture antibiotics for infection control [[2], [3], [4]]. However, the directly released antibiotics and even the species that contain antibiotics in the ecosystem are dangerous and possibly affect human and animal health. Moreover, large amounts of antibiotics released in the surrounding environment can lead to the mutation of antibiotic-resistant microorganisms through food chains [5]. Tetracycline compounds were found at 113.89 mg/L in Shanghai's Huangpu River in 2009 [6]. Antibiotics remain contaminated in Vietnamese urban lakes in 2021. The average sulfonamide content is 117.9 mg/L, and the sulfamethoxazole concentration is 806.5 mg/L [7]. Hence, finding an effective way to remove antibiotics from wastewater is crucial to overcoming these significant issues [8].

Technically, several removal methods, such as ion exchange [9], photocatalytic degradation [10,11], and chemical oxidation [12] are applied for antibiotic disposal. However, these techniques are not economical and environmentally acceptable. Amongst them, the use of porous materials, such as porous carbon, porous silica, porous alumina, zeolite, and metal-organic framework [4,[13], [14], [15], [16]] for adsorption process is considered an inexpensive, effective, and easy technique for removing pollutants from the aqueous solution [17,18]. Furthermore, using porous carbon as an adsorbent for antibiotic removal exhibits notable benefits regarding its high adsorption efficiency and comparatively economical aspects than other adsorbent since porous carbons could be developed from several biomass resources via thermochemical processes such as hydrothermal carbonization, carbonization combined with the activation process [19,20]. Recently, research has focused on using biomass-derived porous carbon to adsorb organic pollutants from wastewater. There is still space for investigation into generating carbon compounds from widely available, cost-effective, environmentally friendly, and sustainable source materials [21]. Many studies have focused on turning biomass waste into carbon materials [22,23]. This waste disposal method is being investigated to reduce raw material costs for producing porous carbon and increase biomass waste usage. Besides agriculture activity and food production often generate a lot of biomass wastes, which can be used as a feedstock for biochemicals and biomaterials [24]**.**

Over the past decades, wastes from aquaculture and aquatic food processing, comprising solid and liquid byproducts, have impacted the ecosystem and caused food production issues. One of those aquatic food processing processes, shrimp processed without the exoskeleton, is exported for high value, generating the overall volume of solid shrimp waste produced by approximately 30–50 % of raw shrimp, depending on the shrimp species. Also, as reported by the Food and Agriculture Organization of the United Nations (FAO), about 3–5 million tons of solid waste are generated annually from the processing of shrimp waste [25]. Typically, shrimp waste including exoskeleton shells, is mainly discarded as bio-waste or used as a low-value plant fertilizer and animal feed [26]. Typically, shrimp shell has a lot of carbohydrates, chitin, and other bio-nutrient components made of organic carbon atoms. Thus, there is considerable interest in exploiting it to make high-value products using innovative technologies. Recent studies reported that shrimp shells use efforts include chitosan and other bioactive chemical synthesis [25,27]. It has also been hydrothermally carbonized into hydrochar with lignite coal-like fuel qualities [28]. Additionally, numerous studies have converted shrimp waste into biochar for usage as a catalytic supporter [29,30], and as an alternate cathode in fuel cells and supercapacitors [31,32]. shrimp waste extract without conversion process has also been evaluated for wastewater heavy metal ion adsorption. Rech et al. (2019) studied metal ion biosorption in natural shrimp shells. Cr^+^ and Fe^+^ ions had maximal adsorption capacities of 62.2 % and 63.4 %, respectively [33]**.** Chitosan from shrimp shell extraction efficiently removed Pb, Cd, Ni, Cu, and Mn from aqueous solution by Abomosallam et al. (2022) [34]**.** Nonetheless, shrimp shell converted by hydrothermal carbonization without activation have poor specific surface area and porosity. Alkali-activated shrimp waste-derived porous carbon had a better porous structure than acid-activated carbon [35,36]. However, KOH-activated raw feedstock had a low specific surface area. Carbonized feedstock used in KOH activation produces SW porous carbon with high porosity [37,38]. Carbonization combined with H_3_PO_4_ activation yielded shrimp waste-derived porous carbon with a specific surface area of 560.6 m^2^/g [39] while Salawu et al. (2022) found it above 2260 m^2^/g [40]. Noticeably, high calcium carbonate concentration in natural shrimp shell structure may reduce pore development. Thus, CaCo_3_ removal pretreatment is necessary for enhancement of pore formation of shrimp waste derived porous carbon.

To the best of our knowledge, the conversion of shrimp waste into nanoporous carbon by using hydrothermal carbonization-assisted KOH activation for removing antibiotics from aquaculture wastewater has yet to be explored elsewhere. Interestingly, hydrothermal carbonization may develop the carbon structure of raw shrimp waste feedstock, which is favorable for chemical activation. Additionally, this study shows the unique ability of hydrothermal carbonization that can be applied to handle any shrimp waste quality in cases where it cannot be further used for chitin or other biomaterial productions into a carbon material. The hydrothermal carbonization of shrimp waste has become a pretreatment process for producing nanoporous carbon.

Therefore, this study is aimed to explore the conversion of shrimp waste (SW) into porous carbon by using hydrothermal carbonization-assisted KOH activation. Moreover, the SW-derived porous carbon was further applied in the adsorption of oxytetracycline, which is selected as an agent of antibiotics used in aquaculture processing. The SW porous carbon was comprehensively studied regarding production yield, porous structure, and other physicochemical characteristics. Further, we employed the SW porous carbon (SWC) with the highest porosity for the experiment of oxytetracycline removal. The isotherm, kinetic, and thermodynamics of oxytetracycline adsorption are investigated. The regeneration of adsorbed SWC was also investigated to study the reusability performance.

## 2 Materials and methods

### 2.1 Materials

Shrimp waste (SW) feedstock was collected from the urban supermarket at Chonburi, Thailand, for utilization as a raw material for nanoporous carbon production. Oxytetracycline (OTC) was purchased from Farm center Co., Ltd., Thailand. The chemicals used in the experiments, including potassium hydroxide (KOH, >85 % purity) and hydrochloric acid (HCl, 37 wt%), were purchased from Carlo Erba reagents (France). High purity-grade (99.99 %) nitrogen was used in all experiments.

### 2.2 Synthesis of shrimp waste nanoporous carbon

**Fig. 1:**
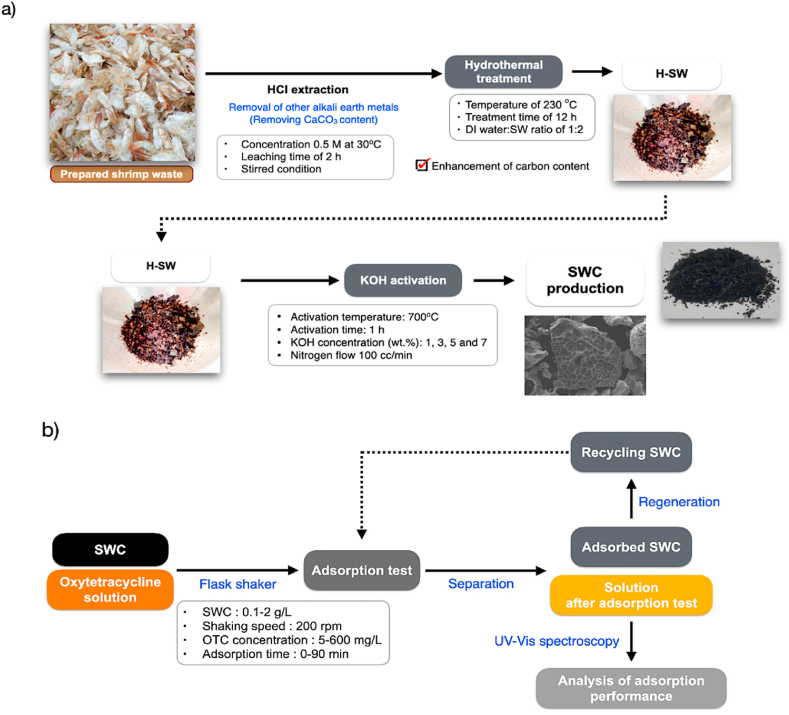
Schematic diagrams of (a) Shrimp waste-derived nanoporous carbon and (b) OTC adsorption process using Shrimp waste-derived nanoporous carbon.

Fig. 1 shows an overview of this study. The SW-derived nanoporous carbon (SWC) was processed as shown schematically in Fig. 1a. However, the ash content referred to inorganic elements of SW feedstock tends to be relatively high due to the nature of shrimp exoskeleton. It is mainly composed of calcium in the form of CaCO_3_. Before the experiments, raw SW was deashed with 0.5 M HCl under a stirred condition of 200 rpm at 30 °C for 2 h. HCl-treated SW was severally rinsed with deionized (DI) water (Milli-Q Ultrapure water system) for adjusting neutral pH, and then, washed SW sample was pretreated by hydrothermal carbonization at 230 °C for 12 h to develop the carbon content for further KOH activation experiments. The hydrotreated SW was dried in an electrical hot air oven (Memmert Universal UF55) at 105 °C for 24 h. Then, dried SW sample was crushed into particle sizes between 0.1 and 0.5 mm. The proximate and ultimate analysis of raw SW, HCl-treated SW (T-SW), and hydrotreated SW (H-SW) are shown in Table 1. H-SW was used as a feedstock to produce nanoporous carbon for activation due to its high carbon content. The H-SW was physically blended with KOH at different 1, 3, 5, and 7 wt% KOH to H-SW, and transferred to the activation process at 700 °C with a holding time of 1 h under the nitrogen atmosphere. The obtained SWCs were washing with 0.5 M HCl, and deionized (DI) water repeatedly to remove the contaminants, and drying at 105 °C for 24 h. The SWC was named by SWC-KOH concentration as SWC-1%KOH, SWC-3%KOH, SWC-5%KOH, and SWC-7%KOH, respectively. Furthermore, SWCs were characterized their physicochemical properties. The optimal SWC with the highest specific surface area and porosity was further selected for the oxytetracycline removal experiment. Moreover, the SWC production yield is expressed in the following equation: (1) based on a dry basis.

(1) $$\text{SWC}\;\text{production}\;\text{yield}\;\left(\%\right)=\frac{{M\text{ass}}_{\text{SWC}}}{{M\text{ass}}_{\text{raw}\;\text{material}}}\times 100$$

**Table 1:** Proximate and ultimate analysis of raw shrimp waste (SW), HCl-treated SW (T-SW), hydrotreated SW (H-SW), and shrimp waste-derived nanoporous carbon (SWC) obtained from different KOH activation.

Properties	Proximate analysis (d.b., %)			Ultimate analysis (d.a.f., %)				
	VM	FC^a^	A	C	H	N	O^a^	S
Raw SW	62.19	6.39	31.42	31.42	4.26	7.97	55.21	1.14
T-SW	82.97	9.44	7.59	45.12	2.18	4.25	47.59	0.86
H-SW	72.95	19.97	6.08	50.93	1.21	1.97	39.45	0.44
SWC-1%KOH	40.28	51.53	8.19	62.71	0.98	1.12	34.68	0.51
SWC-3%KOH	40.97	50.79	8.24	64.42	1.09	0.94	33.18	0.37
SWC-5%KOH	40.14	50.94	8.92	62.19	1.02	0.89	35.48	0.42
SWC-7%KOH	39.87	48.68	11.45	61.57	1.06	0.85	36.44	0.35

d.b.: Dry basis, d.a.f.: Dry-ash-free basis.

a Calculated by different, M: Moisture, VM: Volatile Matter, FC: Fixed Carbon, A: Ash.

### 2.3 Characterization of shrimp waste-derived nanoporous carbon

Thermogravimetric analysis was performed for proximate analysis using NETZSCH TG209, Germany (ASTM D 7582-15; 2015) to determine the moisture, volatile matter, fixed carbon, and ash contents. Ultimate analysis of SW samples for atomic elemental analysis, including carbon, hydrogen, nitrogen, oxygen, and sulfur percentages, were characterized using a CHNS elemental analyzer model LECO Truspec CHN 628. The analysis test procedure was derived from ASTM D 5373-16 (2016) and ASTM D 4239-17 (2017). 100 % of all elements were explicitly subtracted for their oxygen content.

The surface functional chemistry of SWC was evaluated by using transmittance by Fourier Transform Infrared spectroscopy (FT-IR) modeled PerkinElmer Spectrum Two mode at a wavenumber of 4000−400 cm^−1^. The chemical structure of the synthesized SWC was investigated using X-ray photoelectron spectroscopy (XPS) and the Kratos AXIS supra (XPS) surface analysis.

The surface morphology of the obtained SWC was observed by a field emission scanning electron microscope (FE-SEM, Hitachi SU8030, Japan). The SEM operation was conducted at a voltage of 5 kV. Also, the high resolution emission transmission electron microscope (HRTEM) image of the SWC product was studied by using a JEOL JEM-3100F transmission electron microscope operated at an acceleration voltage of 300 kV. The sample was prepared by dispersion with concentrated ethanol solution and then dropped onto the Cu grid (200 square mesh) coated with carbon film.

The textural pore characteristics were measured by N_2_ sorption analysis using a Quantachrome Autosorp iQ-MP-XR at −196 °C. The samples were degassed at 300 °C for 3 h before sorption analysis. A Brunauer-Emmett-Teller (BET) model determined the specific surface area at the relative pressure range from 0.1 to 0.99. Total pore volume was calculated by the Barrett-Joyner-Halenda (BJH) method at the saturated pressure (P/P_0_ = 0.99). The micropore surface area (S_micro_) and micropore volume (V_micro_) were explored using the t-plot method. The pore size distribution was analyzed using the density functional theory (DFT) model.

The graphitic degree, crystallinity and phase composition of the SWC samples were determined using Raman spectroscopy (Thermo Scientific DXR SmartRaman, Germany) and the X-ray diffraction (XRD) model Rigaku SmartLab (Japan), respectively. The X-ray diffractograms were obtained using Cu-Kα radiation at 40 kV and 30 mA, with steps of 0.01° S^−1^ and a step length of 0.5 s over a range of 10° < 2θ < 80° using an X-ray diffractometer.

### 2.4 Oxytetracycline removal experiment

The OTC removal experiments were studied by an adsorption technique using a selected SWC with the highest porosity applied as an alternative sorbent material (Fig. 1b). The adsorption test was carried out by using an orbital shaker-flask clamp platform (TS-520D, Yihder Co., Ltd., Taiwan) operating at a shaking speed of 200 rpm at ambient temperature. The OTC adsorption was carried out with a SWC dosage of 0.4 g/L at different concentrations ranging from 5 to 600 mg/L of OTC solution and an adsorption time of 0–90 min. The regeneration of adsorbed SWC was conducted by a heat treatment process at 400 °C for 2 h under the nitrogen flow adapted from a recent literatures [41,42]. The OTC concentration after the adsorption process was observed by ultraviolet–visible spectrophotometer (T92+ UV–Vis spectrophotometer). The OTC adsorption capacity was calculated at the equilibrium condition and at a given time, q_e_ (mg/g) and q_t_ (mg/g), by the following Equations (2), (3).

(2) $$q_{e}=\frac{\left(C_{i}-C_{e}\right)V}{M}$$

(3) $$q_{t}=\frac{\left(C_{i}-C_{t}\right)V}{M}$$

Where q_e_ and q_t_ are the adsorption capacity of OTC at equilibrium, and time (mg·g^−1^), respectively. C_i_, C_e_, and C_t_ are the initial, equilibrium, and time of OTC concentrations (mg·L^−1^), respectively. V is the OTC solution volume (L), and M is the weight of adsorbent (g) [43].

## 3 Results and discussion

### 3.1 Physicochemical properties of shrimp waste-derived nanoporous carbon

Proximate analysis of raw shrimp waste (SW), HCl-treated SW (T-SW), hydrotreated SW (H-SW), and shrimp waste-derived nanoporous carbon (SWC) is provided in Table 1. Raw SW feedstock has a 6.39 % fixed carbon content, 62.19 % volatile matter, and a relatively high ash content of 31.42 %. High ash content may disturb the pore formation, resulting in a significant decrease in the porosity and specific surface area and a reduction in the adsorption capabilities of the resulting SW-activated biochar. After the HCl washing process, about 75.8 % of the ash content can be removed due to the inorganic soluble leaching mechanism [44]. The HCl treatment may increase the fixed carbon content significantly. The fixed carbon content is specifically modified after hydrothermal treatment before activation. The FC content is elevated to as high as 19.97 %, and the ash content is reduced from 7.59 to 6.08 % due to the dissolving of inorganic substances under severe conditions during HTC treatment [44,45]. However, the amount of fixed carbon present is the most crucial factor in proximate analysis. The SWC nanoporous carbon comprises fixed carbon content between 48.68 and 51.53 %. The presence of ash is in the range of 8.19–11.45 %. An increase in KOH concentration during the activation process significantly affects the reduction of fixed carbon content since the excess of KOH enhances the oxidation degree of carbon atoms in the SWC structure. It is clear from Table 1 that the amount of fixed carbon increases with the severity of the process condition (HTC treatment and activation at high-temperature conditions). This result is due to the thermal decomposition of biomass, which led to the volatilization reaction. However, the proportion of carbon is related to its surface area [46,47]. Moreover, the ultimate analysis of various SW samples is also reported in Table 1. The carbon, hydrogen, nitrogen, oxygen, and sulfur have been measured. According to the ultimate analysis, the carbon percentage of all SW samples is in the direct trend to FC content. The percent carbon of SWC nanoporous carbons is 61.57–64.42 %. The nitrogen content is high due to the nature of shrimp waste. However, the nitrogen and sulfur contents of SW samples are significantly removed after HCl washing and the HTC treatment process through denitrification and desulfurization, respectively. Typically, organic nitrogen and sulfur in several biomass feedstocks have an extremely water-soluble characteristic [44]. Hence, the organic nitrogen and sulfur can be easily diminished by involved water treatment processing.

**Fig. 2:**
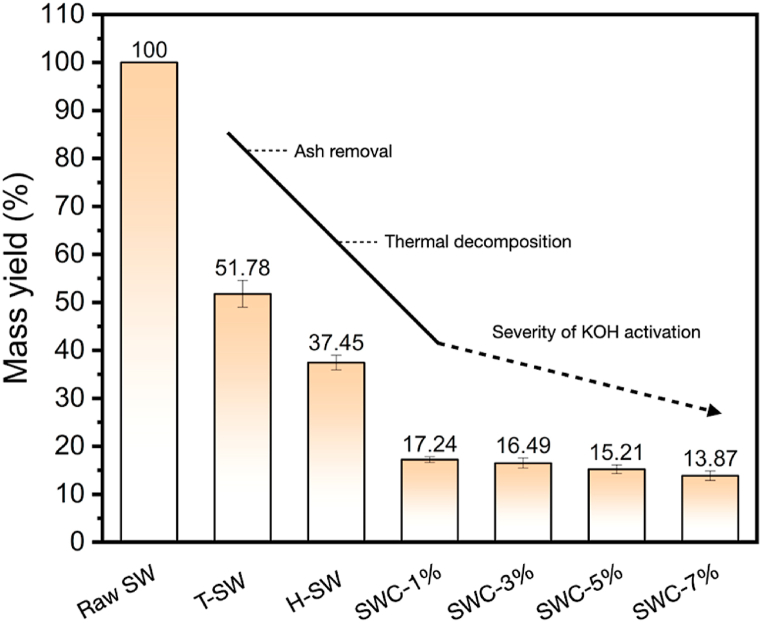
Production yield of shrimp waste (SW) obtained from various processing conditions: HCl-treated SW (T-SW), hydrothermal treated SW (H-SW), and SW derived nanoporous carbon (SWC) at different KOH concentrations.

The production yield results are shown in Fig. 2. They came from different steps in the SWC production process, such as washing with HCl, treating with HTC, and activating with KOH at different concentrations. The mass yield of SW feedstock slowly dropped to 51.78 wt% after the HCl washing treatment process. The ash-leaching process caused this phenomenon. The calcium carbonate in the exoskeleton of shrimp waste is directly decomposed into calcium ions during the HCl washing method. The proximate analyses reported in Table 1 support this conclusion [38]. The HCl-treated SW are subsequently hydrothermally treated at 230 °C for 12 h. The mass yield of H-SW was continually decreased to 37.45 wt%. It was drastically lowered owing to the thermal decomposition reaction during the HTC process. This phenomenon was the case because the thermal breakdown of oxygenated compounds occurs during the HTC process through hydrolysis, dehydration, decarboxylation, and decarbonylation reactions, resulting in the pure carbon of the SW sample before porosity and surface modification. However, the KOH activation was operated at 700 °C for 1 h. This process quite influenced the final production yield of the SWC product. After going through the KOH activation process, the SWC nanoporous carbon production yield was between 13.87 and 17.24 wt%. Seemingly, the increase in KOH concentration has a negative effect on SWC production yield because it increases the activation degree. The carbon structure is more reactive and degrades at a higher KOH concentration [48]. Nonetheless, the surface and porosity of SW nanoporous carbon contributed significantly to a lower overall SWC total production yield.

**Fig. 3:**
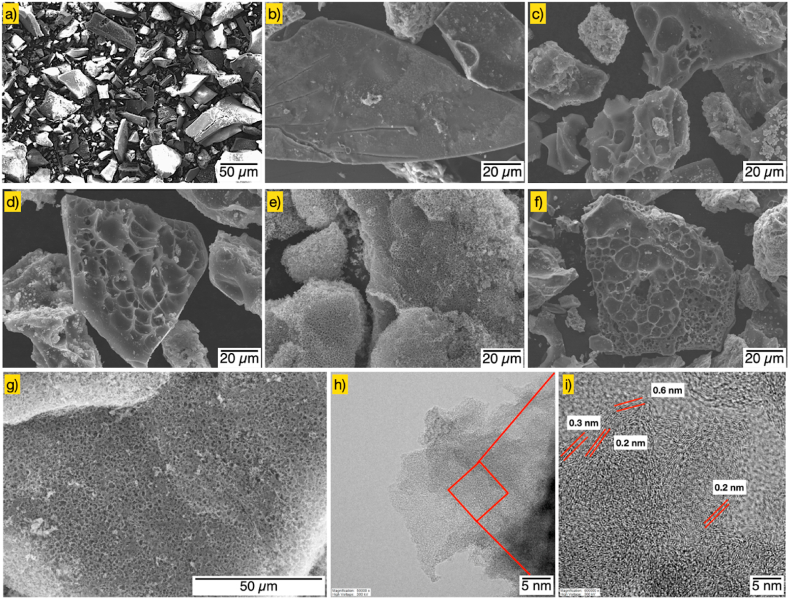
SEM micrographs of (a) T-SW, (b) H-SW, (c) SWC-1%KOH, (d) SWC-3%KOH, (e) SWC-5%KOH, (f) SWC-7%KOH, while high resolution SEM (g), and HRTEM images (h, i) of SWC-5% KOH (the highest S_BET_).

Fig. 3 displays the FESEM and HRTEM images of several SWC nanoporous carbons. In Fig. 3a–f, the observation showed the specific surface characteristics of SW samples, including T-SW, H-SW, SWC-1%KOH, SWC-3%KOH, SWC-5%KOH, and SWC-7%KOH, respectively. No pores or smooth characteristics are observed on the surface of the T-SW and H-SW samples (Fig. 3a and b). This finding also correlated with the pore structure analysis results, showing that the H-SW sample has a relatively low specific surface area of approximately 29.12 m^2^/g. However, as seen on the surface of the SWC (Fig. 3c–f), the pore structures could be observed. As the KOH concentration increased, SWC-5%KOH (see Fig. 3e) showed the highest porosity (the highest BET surface area, listed in Table 2), which reflected a variety of pores. The excess KOH in activation is crucial in diminishing pore formation due to expanding micropores into mesopores [49]. However, the pore cavities observed by FESEM represent the microstructure of the SWC exterior pores. As seen in high-resolution SEM and TEM images of SWC-5%KOH (the highest S_BET_) (Fig. 3g–i), these vast pores on the outside lead into the mesopores and micropores on the inside. The existence of nanoporous structures, both micropores and mesopores, is displayed. The large number of white dots in the carbon matrix in the spaces between the disordered carbon layers suggests the presence of micropores and mesopores in the nanoporous carbon sample. Also, the pore size distribution corresponds to this result, showing that the pore size distribution is in the ultra-micropore range (<1−1.5 nm), as demonstrated in Fig. 5b. Nonetheless, it can be implied that the excellent adsorption performance of OTC from aquaculture wastewater may be obtained by using SWC-5%KOH as an alternative sorbent material.

**Table 2:** Textural pore characteristics of as-prepared SW nanoporous carbons.

Conditions	Pore characteristics				
	S_BET_ (m^2^/g)	V_T_ (cm^3^/g)	V_mic_ (%)	V_mes_ (%)	D_p_ (nm)
H-SW	29.12	0.029	33.57	64.25	5.82
SWC-1%KOH	282.44	0.151	70.36	28.38	1.98
SWC-3%KOH	471.85	0.237	68.95	30.47	2.06
SWC-5%KOH	679.51	0.458	72.62	26.71	2.15
SWC-7%KOH	576.35	0.372	65.21	32.68	2.79

D_p_: Average pore width (4V/A by BET).

**Fig. 4:**
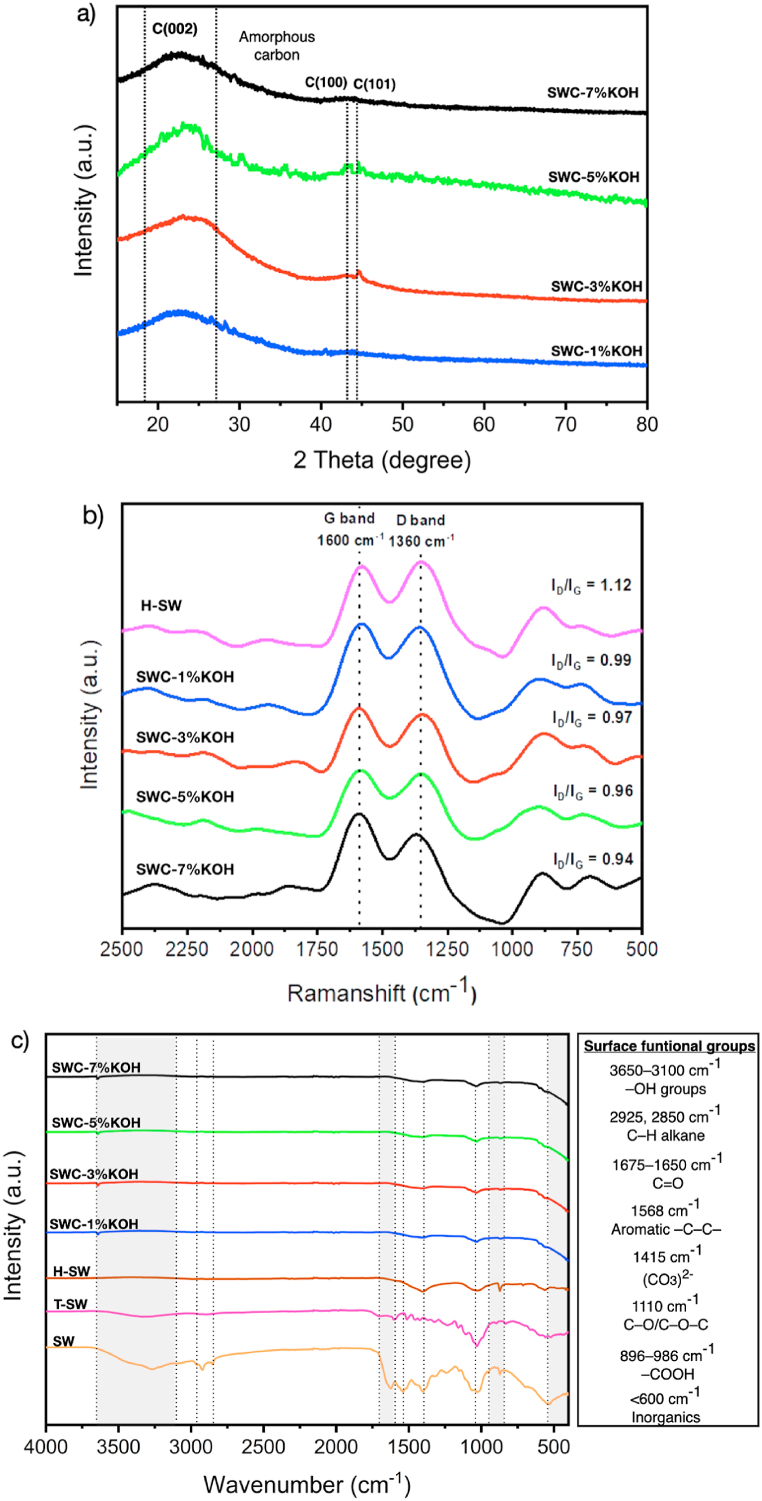
Chemical and structural characteristics of SWC (a) XRD pattern, (b) Raman shift, and (c) FTIR spectra.

**Fig. 5:**
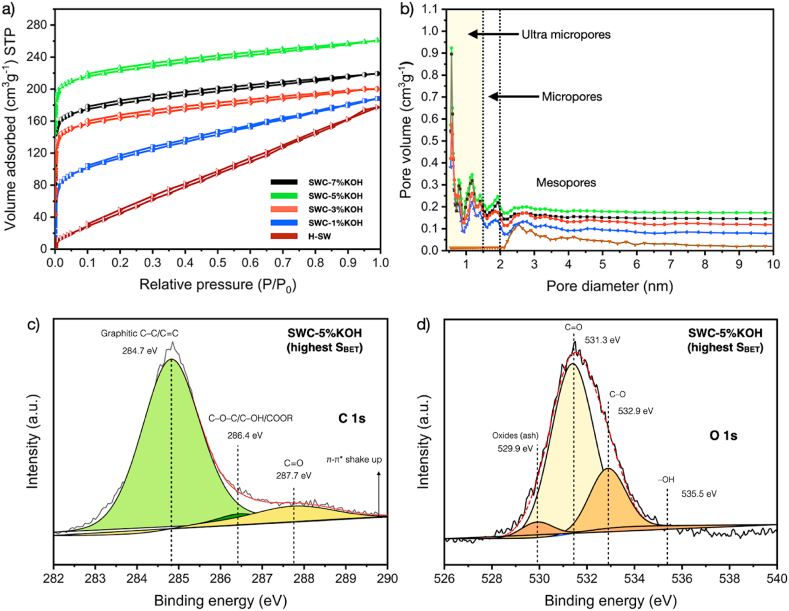
N_2_ adsorption/desorption isotherms (a), pore size distribution, (b) of SW and SWC samples, while XPS spectra including (c) C1s, and (d) O1s spectra of SWC-5%KOH (highest S_BET_).

Fig. 4 shows the chemical and surface structure features of SW samples from a range of process conditions that reveals about the unique properties of SWC samples. In Fig. 4a, the X-ray diffractogram of the SWC is illustrated. These nanostructures were composed of three atomic-plane clusters. The first cluster was observed on the maximum intensity of the X-ray diffractogram with ranges of approximately 15°–26.5° that related to the phase characteristic of amorphous carbon, and the second small intensity peak was located at a similar diffractogram with 2***θ*** of 43°–44.5° is ascribed to graphitic carbon structure in the C (100) and C (101) planes, respectively (following the JCPDS no. 41–1487) [50]. The carbon structure that was observed from SWC samples was amorphous carbon, along with some graphite carbon, as assured by Raman analysis results (Fig. 4b).

As can be seen in Fig. 4b, the Raman spectra of SWC exhibited distinct peaks. These include a G-peak at 1600 cm^−1^ caused by the stretching of the sp_2_ carbon atom and a D-peak at 1360 cm^−1^ caused by a disorder in the amorphous carbon structure. The I_D_/I_G_ intensity ratios of the D-band and G-band were used to determine the degree of graphitization in SWC nanoporous carbons [36]. This finding is the ratio of the structure of graphitic carbon to that of amorphous carbon. The I_D_/I_G_ ratio of H-SW is approximately 1.12, leading to the composition of an amorphous structure. Meanwhile, the I_D_/I_G_ ratios of SWC samples dropped from 1.12 to 0.94 to 0.99, showing that the graphitized structure significantly increased. However, a higher I_D_/I_G_ ratio obtained from SWC provides evidence to enhance the adsorption performance of organic contaminants such as various antibiotics in wastewater, which can also explain the interaction between the two substances. The high graphitic carbon structure of SWC has some potential to serve as an electron donor for the aromatic rings to develop an electron-donor-acceptor relationship between carbon adsorbent and organic molecules based on π-π bond interaction [51]. Moreover, the FTIR spectra of SW and SWC samples are shown in Fig. 4c. The peak intensity ascribed to O–H stretching vibration was located at 3100–3650 cm^−1^, and this intensity peak was reduced, showing that it existed on the surface of SWC in a more stable form due to the elimination of O–H groups during the thermal conversion process [52]. The intense peak of 2850 and 2925 cm^−1^ corresponds to the stretching vibration of the C–H alkane of the chitin structure of raw SW, which significantly decreased after the conversion of raw SW into carbon-rich material using the HTC treatment process. The stretching vibration band between 1675 and 1750 cm^−1^ corresponds to the C=O of aldehydes and ketones, which thermally decompose after SWC production. Meanwhile, the peak intensity of C–C stretching vibration recorded at 1568 cm^−1^ showed existing aromatic compounds in SW samples significantly. This result is due to the thermal conversion of raw SW into carbon nanomaterial, involving both HTC treatment and activation processes that improved the thermal cracking and reforming of volatiles, penetrating deep into the carbon matrix to begin a ring condensation process, and increasing aromatization [53]. The high-intensity peaks at 1010 cm^−1^, and 896–986 cm^−1^were attributed chiefly to carbon chains with carbon-oxygen bonds of C=O and C–O/C–O–C, respectively. These carbon-oxygen structures may refer to both oxygenated compounds in raw SW feedstock and related to the formation of carbon-oxygen bonds during the KOH activation process. The observation for the SWC sample also found the lower intensity of these existing vibration peaks. Furthermore, the peaks at 1415 cm^−1^, and below 600 cm^−1^ were typical CaCO_3_ vibration peaks, which can be obtained at a higher intensity for raw SW [53,54]. This result was consistent with the proximate analyses that revealed the highest ash content of raw SW samples (see Table 1).

The N_2_ adsorption and desorption isotherms of SW samples are displayed in Fig. 5a. The H-SW sorption isotherm curve exhibits the characteristics of a Type II N_2_ adsorption isotherm according to the IUPAC classification [37], indicating a less porous or non-porous structure. This finding means that H-SW has a small, specific surface area. However, the pore structure of SWC nanoporous carbon shows that KOH is an essential part of activating SW [40]. On the other hand, the N_2_ sorption of SWC nanoporous carbon mostly showed the Type I adsorption isotherm, which means that the SWC structure has micropores [40]. SWC-5%KOH has the highest N_2_ adsorbed volume. This result is due to its huge surface area and porosity, as seen in Table 2 (S_BET_ of 679.51 m^2^/g, and V_total_ of 0.458 cm^3^/g). Moreover, the pore size distributions of all SW samples are displayed in Fig. 5b. Adsorption efficiency and selectivity are affected by the size distribution of pores, which varies depending on the source material [55]. While micropores in porous materials may adsorb tiny molecules less than 2 nm, mesopores such as dye and heavy organic compounds can adsorb larger contaminant molecules more significantly than 2 nm. The determination of the pore size distribution was analyzed using the DFT method. The results showed that the significant pore size was in the range of micropore size (<2 nm).

All SWC nanoporous carbons have a heterogeneous micropore size distribution ranging from 0.2 nm to 1.5 nm with four distinct peaks, demonstrating a significant characteristic of the ultra-micropore size distribution of SWC nanoporous carbons. As observed from the highest peak of each SWC product, most of the micropores had a diameter of approximately 0.5 nm and a pore volume range of between 0.56 cm^3^/g and 0.92 cm^3^/g. The samples also contain three minor peaks at 0.9, 1.2, and 1.4 nm, indicating that the pore volume is between 0.12 and 0.26 cm^3^/g. As seen in Fig. 5b, mesopores were also found between 2.2 and 5.8 nm for all SW samples, including H-SW. For H-SW, the pore size distribution appears to feature a broad size distribution of around 2.2–3.4 nm, indicating that mesopores and external pores exist. However, the existing mesopores and external pores significantly reduce porosity and specific surface area (Table 2) [56]. Meanwhile, SWC exhibited mostly narrow mesopores with a sharp peak size of around 2.5 nm. An increased KOH concentration significantly leads to a higher proportion of mesopore size distribution, reducing the specific surface area of SWC. It may be inferred that KOH activation results in large pore cavities, including mesoporous structures [57]. For the SWC samples, pore volume is also related to surface area.

The textural pore structure of SW samples is shown in Table 2. It showed the average pore diameter (D_avg_), specific surface area (S_BET_), total pore volume (V_total_), micropore volume (V_micro_), and mesopore volume (V_meso_). The S_BET_ of H-SW increased from 29.12 m^2^/g to 679.51 m^2^/g after KOH activation. It might be ascribed to the presence of exposed pores caused by the volatile emission of the SW matrix and the presence of KOH as an activating agent during high temperature processing [57]. However, an increase in KOH concentration to the KOH excessive condition (SWC–7%KOH) significantly decreased overall pore volume and specific surface area due to the high degree of oxidation between KOH and carbon, leading to the reduction of overall carbon elemental content and caused the expansion of pore size [58]. The findings were supported by establishing the elemental composition listed in Table 1.

In Fig. 5c and d, XPS analysis was used to measure the chemical bonding information for the SWC-5%KOH. To determine their chemical bonding, high-resolution XPS spectra on the C1s and O1s areas were recorded and painstakingly deconvoluted. The C1s and O1s were presented in the XPS survey spectra. The high-resolution XPS peak of C1s is involved three components (Fig. 5c). The first intense peak located around 284.7 eV was attributed to the sp_2_ bond of graphitic C–C and C]C that is ascribed to the graphite structure in the SWC nanoporous carbon framework [59]. The Raman analysis obviously supported these findings. The remaining two component peaks at higher binding energies are typically attributed to the sp_3_ bond of C–O–C, C–OH, and COOR structures, which correlated to carbon atoms bonded to oxygen atoms at binding energy approximately of 286.4 eV and C=O were responsible for the peak at 287.7 eV [35]. Notably, the formation of π-π* shake-up appeared at the existing broad peak 289.8 eV, which assured the appearance of graphitization. This observed peak appeared due to the high-temperature pyrolysis operation [8,36]. As seen in Fig. 5d the XPS O1s spectra were deconvoluted into four component peaks, including stretching of metal oxides in SWC ash content (529.9 eV), C=O in carbonyl and carboxyl groups (531.3 eV), C–O in epoxides (532.9 eV), and the adsorbed moisture (535.5 eV), respectively [60].

According to the characterization of SW-derived nanoporous carbon, the SWC-5%KOH revealed specific properties such as the highest S_BET_, excellent porosity, graphitic carbon characteristic, and special surface chemistry, which could be applied as an efficient bio-adsorbent for oxytetracycline (OTC) removal from wastewater. Typically, the specific surface area and porosity of any adsorbent materials directly result in the adsorption capacity. Furthermore, some surface characteristics also have an interesting effect on adsorption performance. Regarding the surface chemistry of adsorbent, the presence of some functional groups on porous carbon surfaces promotes the adsorption of OTC. XPS spectra reveal the presence of oxygen-containing functional groups on the SWC surface, which may create H-bonds, as well as the presence of hydroxyl and amino groups in OTC, which can also form H-bonds that can improve the OTC adsorption capacity [18,51]. However, the adsorption test for OTC was carried out, and the adsorption performance was explained using shrimp waste-derived nanoporous carbon (SWC–5%KOH) in the next session.

### 3.2 Adsorption of oxytetracycline using shrimp waste nanoporous carbon

#### 3.2.1 Adsorption isotherms

**Fig. 6:**
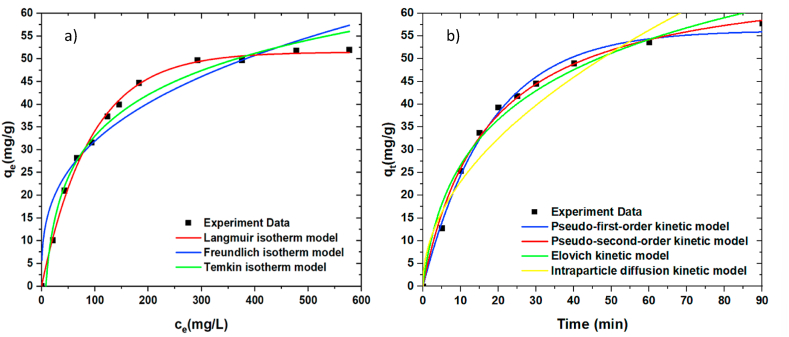
(a) Adsorption isotherms of OTC using SWC; adsorption time of 60 min at different concentration of 5–600 mg/L, and (b) Adsorption kinetic curves for OTC adsorption onto SWC surface; c_i_ = 300 mg/L at ambient temperature with different adsorption times of 0–90 min (SWC dosage of 0.4 g).

The adsorption isotherm of SWC-5%KOH was evaluated at an adsorption time of 60 min at a variety of OTC concentrations in the range of 5–600 mg/L using a SWC dosage of 0.4 g/L. The exploration of the adsorption mechanism of OTC on the SWC surface was the purpose of the adsorption isotherm that was carried out. The adsorption isotherm of OTC was studied using three distinct adsorption isotherm models, including the Langmuir, Freundlich, and Temkin isotherm models, in order to fit the equilibrium isotherm data, and the fitting curves for all three models are depicted in Fig. 6a. The maximal adsorption capacity is represented by the Langmuir isotherm model for monolayer adsorption on the SWC surface. As shown in Equation (4), the adsorbate molecules were supposed to adhere to specific places, with one molecule fitting into each site. As shown in Equation (5), the Freundlich isotherm reflected multi-characteristic adsorption mechanism on the SWC surface via multi-site intermolecular interactions, resulting in a multilayer adsorption and non-linear model. Moreover, the Temkin isotherm model, which depicts the effects of some indirect adsorbent/adsorbate interaction on the adsorption isotherm, is especially considered. Because the heat of adsorption depends on the temperature of all the molecules in the layer, the model predicts that as coverage increases, the heat of adsorption will fall linearly rather than logarithmically. The Temkin isotherm was investigated using Equations (6), (7).

(4) $$q_{e}=\frac{q_{m}k_{L}C_{e}}{1+k_{L}C_{e}}$$

Where q_e_ is the OTC adsorption capacity, q_m_ is the maximum adsorption capacity corresponding to the monolayer adsorption, C_e_ is the OTC concentration at equilibrium condition, and k_L_ is the Langmuir constant [61].

(5) $$q_{e}=k_{F}C_{e}^{\frac{1}{n}}$$

Where the constants k_F_ value, and n depend on the interaction adsorbent-solution and the adsorption temperature. However, 1/n value may be less or greater than unity. The 1/n value is below the unity showing that favorable adsorption is occurred, while 1/n value below one, a normal adsorption occurs. Cooperative adsorption is occurred in the case of 1/n being above one [62].

(6) $$q_{e}=B\;\ln\;K_{T}+B\;\ln\;C_{e}$$

(7) $$B=\frac{\text{RT}}{b}$$

Where K_T_ is Temkin isotherm equilibrium binding constant (L·g^−1^); b is Temkin isotherm constant; R is universal gas constant (8.314 J mol^−1^ K^−1^); T is the temperature at 298 K; B is constant related to the heat of sorption (J·mol^−1^) [62].

In the OTC adsorption test, the finding results showed that only the correlation coefficient value (R^2^) is significant when compared to the other three fitting values. According to the magnitude of the correlation coefficient, it appears that the results for OTC adsorption onto SWC are most closely aligned with the Langmuir isotherm (R^2^ = 0.993), as listed in Table 3. The Langmuir isotherm is a model of monolayer adsorption that is commonly used and is appropriate for use when attempting to describe an adsorption process that does not include intermolecular interaction. The highest adsorption capacity (q_m_) that was calculated for OTC was approximately 61.259 mg/g. However, the adsorption capacity commonly depends on the adsorbent characteristics, including surface area, surface functional chemistry, and pH of the adsorbent [63]. The SWC nanoporous carbon is strongly aromatic and has the ability to interact with OTC molecules [64]. The SWC nanoporous carbon is strongly aromatic and has the ability to interact with OTC molecules [33,64]. In return, OTC may be able to interact with adsorbents by hydrogen bonds in the SWC matrix. In addition, OTC is an ionizable molecule that can form strong electrostatic interactions with adsorbents [65]. Also, the porosity possesses high surface areas, and porosity shows great capability of adsorptive performance. Hence, surface area and porosity are significant variables in adsorption approaches.

**Table 3:** Adsorption isotherm parameters OTC using SWC adsorbent.

Model	Parameter	OTC
Langmuir isotherm model $q_{e}=\frac{q_{m}k_{L}C_{e}}{1+k_{L}C_{e}}$	q_m_ (mg/g)	61.259
	k_L_ (L/mg)	0.012
	R^2^	0.993
Freundlich isotherm model $q_{e}={\;k}_{F}C_{e}^{\frac{1}{n}}$	$k_{F}$	6.828
	1/n	0.334
	R^2^	0.898
Temkin isotherm model $q_{e}=B\;\ln\;K_{T}+B\;\ln\;C_{e}$ ; $B=\frac{\text{RT}}{b}$	B	13.130
	$K_{T}$	0.123
	R^2^	0.982

*T(K) is the temperature in Kelvin; R is the ideal gas constant (8.314 J mol^−1^ K^−1^); K_L_, K_F_, n, B, K_T_, k and b are the constants for the corresponding formula, respectively.

#### 3.2.2 Adsorption kinetics

In Fig. 6b, the adsorption kinetic was used to investigate the OTC removal rate and identify the adsorption mechanism by using SWC-5%KOH. The kinetic experiments were performed by using an initial OTC concentration of 300 mg/L, the SWC dosage of 0.4 g/L, the ambient temperature of 30 °C, and the adsorption contact time of 5–90 min as the quantities considered for the operational variables. For adsorption kinetic study, numerous kinetic models, including pseudo-first-order and pseudo-second-order, are applied to explain the adsorption mechanisms of a pollutant on an SWC surface. The pseudo-first-order and pseudo-second-order kinetic models were expressed as follows: Equations (8), (9), respectively.

(8) $$Q_{t}=q_{e}\left(1\;–\;\exp^{‐k_{1}t}\right)$$

Where q_e_ and q_t_ are the amounts of sorbate uptake per mass of sorbent at equilibrium and at any adsorption time (min), respectively, while k_1_ (min^−1^) is the rate constant of the pseudo first-order equation [62].

(9) $$Q_{t}=\frac{q_{e}^{2}k_{2}t}{q_{e}k_{2}t+1}$$

Where q_e_ (mg/g) and q_t_ (mg/g) are the sorbate amount adsorbed at equilibrium and any adsorption time (min), respectively and k_2_ (mg·g^−1^·min^−1^) is the pseudo second order equation constant rate [62].

For extra investigation on OTC adsorption kinetic on the SWC surface, the Elovich kinetic model was applied to understand the chemisorption phenomena of OTC adsorbed on the SWC surface. The Elovich kinetic model is expressed by Equation (10). However, the intraparticle diffusion kinetic models were applied to consider pore diffusion, which is proposed as follows in Equation (11). The intraparticle diffusion kinetic model involves a mass transfer of adsorbate (film diffusion), surface diffusion, and pore diffusion, which assumes the internal diffusion of the sorbate is the slowest step, resulting in the rate-controlling step during the adsorption process and that adsorption is instantaneous [65].

(10) $$Q_{t}=\frac{1}{\beta }\ln\left(1+\alpha \beta t\right)$$

Where q_t_ is the amount of sorbate (mg·g^−1^) at a particular time. Meanwhile, α represents the initial adsorption rate (mg·g^−1^·min^−1^). β is the extent of surface coverage (g·mg^−1^), and the process activation energy [66].

(11) $$Q_{t}=k_{\text{diff}}t^{\frac{1}{2}}+C$$

Where C (mg/g) is the intercept, and k_diff_ (mg·g^−1^·min^−1^) is the intraparticle diffusion rate constant.

The adsorption kinetics was carried out to explore the adsorption mechanism of OTC on the SWC surface. The kinetics model data were fitted with the Pseudo first-order kinetic model, Pseudo second-order kinetic model, Elovich kinetic model, and intraparticle particle diffusion kinetic model, respectively. The fitting curves for all four models are shown in Fig. 6b. As compared with the four fitting results, The OTC removal efficiency predicted by the Pseudo-second-order kinetic agrees well with the adsorption experimental data with a higher R^2^ of 0.995, as seen in Table 4. In general, the pseudo-second-order model is more commonly employed to represent chemisorption than physisorption processes. As a result, the exchange of electrons or valency between the OTC and SWC was not insignificant throughout the adsorption process [67]. These results further confirmed that the adsorption mechanism of OTC on SWC is particularly chemical adsorption. It can be implied that the hydrogen bonding between the hydroxyl groups in OTC molecules and the oxygen-containing groups on the SWC surface, as well as electron-donor-acceptor interactions, are specifically recognized to be responsible for the rapid adsorption of OTC antibiotics [34,68]. Moreover, the rate-limiting processes in OTC adsorption were identified as an intraparticle diffusion kinetic model [68]**.**

**Table 4:** Adsorption kinetic parameters of OTC adsorption onto SWC surface.

Model	Parameter	TC
Pseudo-first-order kinetic model $q_{t}=q_{e}\left(1\;‐\;e^{‐k_{1}t}\right)$	k_1_ (min^−1^)	0.057
	q_e_ (mg·g^−1^)	56.220
	R^2^	0.993
Pseudo-second-order kinetic model $q_{t}=\frac{q_{e}^{2}k_{2}t}{q_{e}k_{2}t+1}$	k_2_ (min·mg·g^−1^)	0.001
	q_e_ (mg·g^−1^)	69.339
	R^2^	0.995
Elovich kinetic model $q_{t}=\frac{1}{\beta }\ln\left(1+\alpha \beta t\right)$	α	6.324
	β	0.058
	R^2^	0.979
Inter-particle diffusion kinetic model $q_{t}=k_{\text{diff}}t^{\frac{1}{2}}+c$	k_diff_ (mg·g^−1^ min^−1/2^)	7.263
	c (mg·g^−1^)	3.019
	R^2^	0.904

Note: k_1_, k_2_, α, and β are the constants for the corresponding formula, respectively.

#### 3.2.3 Adsorption thermodynamics

Adsorption thermodynamic studies were performed at different temperatures in the range of 298–328 K at a constant condition of the initial OTC concentration of 300 mg/L, the SWC dosage of 0.4 g/L, and the adsorption period time of 60 min. In this research, thermodynamic parameters such as variation in enthalpy (ΔH), Gibbs free energy change (ΔG), and entropy alteration (ΔS) were determined by using equations (12), (13), (14) as follows:

(12) $$\Delta G=‐\text{RT}\ln\;K_{d}$$

(13) $$K=\frac{qe}{Ce}$$

(14) $$\ln\;K=\frac{\Delta G}{\text{RT}}=-\frac{\Delta H}{\text{RT}}+\frac{\Delta S}{R}$$

Where ΔG°, ΔH°, and ΔS° are the Gibbs free energy (kJ·mol^−1^), enthalpy (kJ·mol^−1^), entropy (kJ·mol^−1^ K^−1^). T is the absolute temperature of system (K), and R is the gas on stent 8.314 J mol^−1^ K^−1^. K is the distribution coefficient which can be determined through the ratio of q_e_ (mg·g^−1^) to c_e_ (mg·L^−1^).

**Fig. 7:**
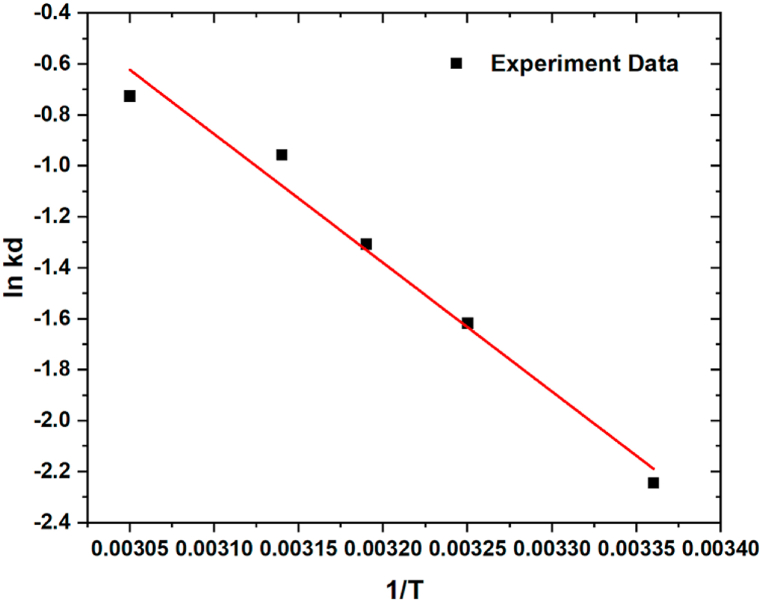
The plot of ln K_d_ vs 1/T for the approximation of thermodynamic constraints for OTC adsorption onto SWC using dosage of 0.4 g/L; c_i_ = 300 mg L^−1^ time 60 min for oxytetracycline solution.

The plot of ln K_d_ versus 1/T for the approximation of thermodynamic constraints for OTC adsorption by SWC-5%KOH using a constant condition as follows: dosage of 0.4 g/L; c_i_ = 300 mg/L time 60 min for oxytetracyclines solution are displayed in Fig. 7. Nevertheless, Table 5 shows the calculated changes of the enthalpy, entropy, and Gibbs free energy displayed these values of the thermodynamic parameters for the OTC adsorption by using SWC. It can be seen the occurrence of positive enthalpy (ΔH) values revealed that the adsorption of OTC was significantly an endothermic characteristic. This result is good and agree with recent studies [29,65]. Also, the values of Gibbs Free Energy (ΔG) at different adsorption temperatures between 298 and 328 K were −36.596, −37.825, −38.440, −39.055, and −40.284 kJ mol^−1^, respectively. As seen in Table 5, the ΔG values dropped gradually as the adsorption temperature increased, suggesting that the degree of spontaneity of the reaction rises and that higher temperatures aid in the adsorption of OTC. Additionally, the value ΔG implies that the adsorption process occurred spontaneously. A positive value of ΔS indicates that the degree of disorder at the solid-liquid interface grows during the adsorption process. Moreover, a positive value of adsorption ΔH ascribed to randomness at the solid-liquid interface during the adsorption process, indicating that the adsorption process was an endothermic reaction pathway [12,67,68].

**Table 5:** Calculated Changes of the Enthalpy, Entropy, and Gibbs Free Energy of adsorption thermodynamic of OTC adsorption using SWC.

Temperature (K)	ΔG (kJ·mol^−1^)	ΔH (kJ·mol^−1^)	ΔS (kJ·mol^−1^)
298	−36.596	42.00233	122.94743
308	−37.825		
313	−38.440		
318	−39.055		
328	−40.284		

#### 3.2.4 Effect of SWC dosage on OTC adsorption performance

**Fig. 8:**
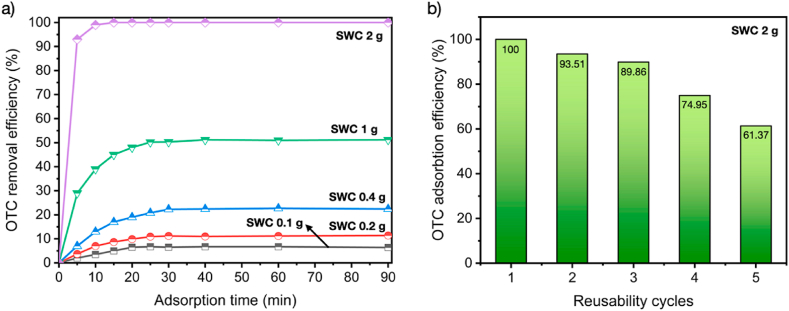
OTC adsorption performance (a) Effect of SWC dosage on the OTC adsorption performance and (b) Reusability of SWC using a constant OTC concentration of 300 mg/L.

Fig. 8 shows the OTC adsorption efficiency using the optimal SWC (SWC–5%KOH). The influence of SWC quantity used for OTC adsorption was evaluated by applying different SWC dosages of 0.1–2.0 g/L for maximizing OTC adsorption performance, as illustrated in Fig. 8a. The OTC adsorption was performed in the shaker unit with 250 mL OTC solution at a concentration of 300 mg/L. The adsorption performance was directly calculated by using UV-VIS spectroscopy to determine the concentration of OTC aqueous solution after an adsorption period of 90 min. In these adsorption experiments, shaking conditions play a significant role in increasing the rate of absorption [69]. The unit adsorption capacity peaked at 99.98 % when the dose of SWC-5%KOH was raised to 2 g after 15 min of adsorption time, and it steadily declined as the dosage was decreased. The OTC adsorption rate was definitely elevated, along with the SWC dosage. The adsorbed amount was dropped oppositely, indicating that the vacant pores or unabsorbed surfaces of SWC were continually lower after the beginning of OTC adsorption. This finding was linked to the availability of a greater number of unoccupied surface sites and a larger surface area with the rising dosage, which resulted in an increase in the removal rate of OTC [69]. Also, the finding result shows that SWC exhibited excellent OTC adsorption performance between 6.85 % and 99.98 %, depending on the existing SWC dosage of 0.1–2.0 g/L during the OTC adsorption test. These findings also showed that the reduction of adsorption time can be reached by increasing the sorbent quantity or using the excess sorbent dosage. Moreover, the reusability test was successfully investigated using the SWC dosage of 2 g/L for complete OTC adsorption. The regeneration of SWC adsorbent is significant to meet practical uses. The adsorption efficiency slightly decreased from 100 % to 89.86 % after the 3 cycles of adsorbed SWC regeneration. Moreover, the OTC adsorption was decreased to 61.37 % after 5 cycles, indicating that the regeneration using a heat treatment significantly generated the soot particles from adsorbed OTC molecules covering the SWC pore structure [70], as well as the high temperature during regeneration may impact the pore degradation between carbon matrix and OTC adsorbate [71], resulting in the continuously lowering OTC adsorption efficiency after several regenerations, as displayed in Fig. 8b.

## 4 Conclusions

The nanoporous carbon derived from shrimp waste (SWC) is effective in removing the antibiotic oxytetracycline (OTC) from an aqueous solution. The high porosity and specific surface area of SWC substantially increase the OTC adsorption capacity. SWC has the highest pore structure after activation with 5%KOH, with a specific surface area of 679.51 m^2^/g, and a total pore volume of 0.458 cm^3^/g. The SWC dosage had a considerable impact on OTC adsorption performance. At the SWC dosage was around 2 mg/L, the adsorption performance was the best, with the greatest adsorption OTC performance of 99.98 %. The Langmuir model best matches the adsorption isotherms data, whereas the pseudo-second-order model better represented the adsorption kinetics data. Moreover, the Gibbs free-energy changes varied from −40.284 to −36.596 kJ/mol, and the enthalpy change was 42.00233 kJ/mol, showing that the adsorption of OTC by SWC constituted an endothermic process. The OTC adsorption performance ranged from 100 % to 61.37 % after the 5 cycles of regenerating the adsorbed SWC. Meanwhile, the intraparticle diffusion and OTC adsorption were found to be rate-limiting stages. According to the findings, the improvement in the adsorptive performance of SWC nanoporous carbon could be achieved by optimizing operating parameters in the activation process and fine-tuning the concentration of the KOH activating agent or investigating other activating agents during the SWC production. The approach to use waste from shrimp processing for producing bio-adsorbents for the removal of oxytetracycline and other antibiotics contained in aquaculture wastewater offers an alternative concept in green technology to sustainable aquaculture systems.

## Availability of data and materials

The authors do not have permission to share data.

## CRediT authorship contribution statement

**Napat Kaewtrakulchai:** Writing – original draft, Investigation, Funding acquisition, Conceptualization. **Nippit Samattakarn:** Methodology, Conceptualization. **Sirayu Chanpee:** Writing – original draft, Visualization, Validation. **Pornsawan Assawasaengrat:** Writing – review & editing. **Kanit Manatura:** Writing – original draft. **Sutthipoj Wongrerkdee:** Writing – review & editing, Formal analysis. **Apiluck Eiad-Ua:** Writing – review & editing, Validation, Supervision, Methodology.

## Declaration of competing interest

The authors declare that they have no known competing financial interests or personal relationships that could have appeared to influence the work reported in this paper.
